# Cardiac markers in children and adolescents at the onset of type 1 diabetes mellitus

**DOI:** 10.1371/journal.pone.0342397

**Published:** 2026-02-09

**Authors:** Sara Musterer, Mandy Vogel, Wieland Kiess, Sandy Richter, Heike Bartelt, Christof Meigen, Uta Ceglarek, Anja Willenberg, Ronald Biemann, Thomas Kapellen, Alexandra Kiess

**Affiliations:** 1 Leipzig University, Medical Faculty, University Hospital for Children and Adolescents Leipzig, Center for Pediatric Research (CPL), LIFE Child, Leipzig, Germany; 2 German Center for Child and Adolescent Health (DZKJ), partner site Leipzig/ Dresden, Leipzig, Germany; 3 Pediatric Research Center, Leipzig University, University Hospital for Children and Adolescents, Department for Child and Adolescent Medicine, Leipzig, Germany; 4 University Hospital for Children and Adolescents Leipzig, Leipzig, Germany; 5 Institute for Laboratory Medicine, Leipzig University, Leipzig, Germany; 6 Department of Child and Adolescent Medicine, Pediatric Cardiology Section, University Hospital Jena, Jena, Germany; University of Dundee, UNITED KINGDOM OF GREAT BRITAIN AND NORTHERN IRELAND

## Abstract

**Background:**

The incidence of type 1 diabetes mellitus (T1DM) in children and adolescents is rising, and both micro- and macrovascular complications pose significant health risks. For ketoacidosis or fluctuations in blood glucose, myocardial injury may occur and lead to severe complications. Cardiac markers such as high-sensitivity troponin T and N-terminal pro-b-type natriuretic peptide (NT-proBNP) are used to detect cardiovascular damage in adults but have been explored less in pediatric populations with T1DM.

**Objective:**

We aimed to investigate the relationships between high-sensitivity troponin T and NT-proBNP levels at T1DM onset and their associations with metabolic markers (Hemoglobin A1c, pH, and glucose) to identify potential early markers of myocardial damage in children with diabetes.

**Methods:**

306 children and adolescents with newly diagnosed T1DM were enrolled from our hospital, while a matched control group of 1,259 healthy children was drawn from the LIFE Child study. Blood samples were analyzed for high-sensitivity troponin T and NT-proBNP concentrations. Descriptive statistics and censored regression models were used to assess associations between biomarkers and metabolic parameters.

**Results:**

Children with recent T1DM had significantly higher high-sensitivity troponin T levels and lower NT-proBNP levels than the control group. High-sensitivity troponin T levels were positively correlated with Hemoglobin A1c in the T1DM group, while NT-proBNP levels were negatively associated with the pH value. For blood glucose levels, a significant correlation was found between blood glucose and high-sensitivity troponin T, but no association was observed with NT-proBNP. There was a significant association between weight change during hospitalization and high-sensitivity troponin T in female patients.

**Conclusion:**

Elevated high-sensitivity troponin T and reduced NT-proBNP levels at T1DM onset suggest early myocardial stress in affected children. These cardiac markers, in conjunction with metabolic parameters, could serve as potential early indicators of cardiovascular risk in this population.

## Introduction

### Background

The incidence of type 1 diabetes mellitus (T1DM) in children and adolescents is increasing [1,2]. Micro- and macrovascular complications of the disease are potentially life-threatening and, therefore, should be identified and treated early. Both in the case of ketoacidosis as an acute complication and as a long-term consequence of hyperglycemia, vascular changes can lead to myocardial injury and thus to severe complications such as heart failure or sudden cardiac arrest [3–5]. To detect cardiovascular damage in general, cardiac markers such as troponin and N-terminal pro brain natriuretic peptide (NT-proBNP) are used regardless of diabetes in adults and in children [6,7]. In the pediatric population, cardiac biomarkers such as troponin and NT-proBNP have shown promising validity and clinical significance for the early detection of myocardial injury, though their interpretation requires age-specific reference ranges and a clinical context [8,9].

Previous smaller studies have indicated that ketoacidosis and hypoglycemia can lead to myocardial injury [3,4,10]. Such changes can be detected through elevated levels of troponin and NT-proBNP and cardiac imaging [11–15]. Case reports have shown that children and adolescents, especially at the onset of diabetes with diabetic ketoacidosis, can have higher values for troponin and may have an increased cardiac risk [16–18]. However, the risk factors, extent of damage, and detection methods are still largely unclear.

### Aims and objectives

The aim of this study was to investigate how high-sensitivity troponin T (hsTnT) and NT-proBNP levels are related to metabolic markers at the time of T1DM onset as a way to provide an early identification of patients at risk for cardiac damage.

## Materials and methods

306 children and adolescents aged 0–19 years were included at the initial clinical onset of T1DM in our hospital. Study inclusion occurred according to the diagnostic ISPAD criteria for childhood diabetes for children and adolescents – ISPAD Clinical Practice Consensus Guidelines 2006–2007. Presentations with DKA as well as non-DKA presentations characterised by typical symptoms (polyuria, polydipsia, weight loss) were enrolled. The Diabetes Center for Children and Adolescents at the Children’s Hospital of the University of Leipzig is part of the Saxon Childhood Diabetes Registry, which has been collecting data on children and adolescents with type 1 to type 3 diabetes mellitus between 1999 and 2023. With the consent of the patients and parents, the data are stored in the registry, and blood samples collected at the time of onset and during follow-up checks are frozen and stored for later examination [2]. The Childhood Diabetes Registry of Saxony was approved by the Ethical Committee of the Medical Faculty of the University of Leipzig (Reg. Nr. 236/21-ek).

A sex-, age-, and BMI-SDS-matched control group of healthy children and adolescents was drawn from the LIFE Child study [19]. The LIFE Child Study was designed in conformity with the Declaration of Helsinki and its later amendments. It is registered with ClinicalTrial.gov (NCT02550236) and the study protocol was approved by the Ethical Committee of the University of Leipzig (Reg. No. 264-10-19042010). Written informed consent was obtained from all parents and children. Body height and weight, medical history, and blood samples were assessed during visits in accordance with standardized protocols. Blood samples were collected in a fasting state [19,20].

Data collection for this study was initiated on 27/03/2023 for the Saxon Childhood Diabetes Registry and on 20/10/2023 for the LIFE child study.

Data collection and analysis were conducted separately. Before any analyses began, the first author filled in missing data and had access to participants’ health records, a process that was supervised by the responsible hospital staff. Once this process was completed, access to the records was revoked. The data was then pre-processed and pseudonymized in accordance with data protection laws by the data management team of the Medical Faculty, without any involvement from the authors. The pseudonymized dataset was subsequently provided to the first author for analysis. The authors did not have access to any identifiable data from the control group. Only the first author (SM) and the statistician (MV) had access to the processed research dataset.

Blood samples were collected as specified by the study protocol and stored at the Leipzig Medical Biobank. Serum concentrations of hsTnT and NT-proBNP were measured at the Institute of Laboratory Medicine, Clinical Chemistry, and Molecular Diagnostics, University of Leipzig, following the manufacturer’s instructions. For both the LIFE Child and T1DM cohorts, the analyses were performed on an automated Cobas 8000 e801 laboratory analyzer (Roche Diagnostics, Mannheim, Germany) using an electrochemiluminescence immunoassay (ECLIA) based on the sandwich principle (Roche Diagnostics, Mannheim, Germany). The primary measurement range for the NT-proBNP assay was 5–35,000 ng/L. The primary measurement range for the hsTnT assay was 3–10,000 ng/L. The hsTnT values from the LIFE Child cohort prior to 2018 were measured using the Cobas 8000 e602 analyzer, with a primary measurement range of 5–10,000 ng/L. Comparison measurements between the two assays showed very good accordance. The assessment of additional laboratory parameters (HbA1c, glucose, and pH) was conducted in accordance with the clinical standards of the Institute of Laboratory Medicine, Clinical Chemistry, and Molecular Diagnostics at the University of Leipzig.

Descriptive statistics are presented as medians and interquartile ranges for continuous variables and as counts and percentages for categorical variables. HsTnT and NT-proBNP were transformed into sex- and age-adjusted standard deviation scores (SDS) using references from Kiess et al [20]. Values below the detection limit and the corresponding SDS were marked as left-censored.

Associations between the cardiac markers hsTnT-SDS and NT-proBNP-SDS and Hemoglobin A1c (HbA1c), pH, glucose, and weight change during hospitalization (in %) as well as group differences (location and variance) were assessed using censored regression, assuming a censored normal distribution of SDS for hsTnT and NT-proBNP. We performed analyses adjusted for both BMI and pH, which did not alter the observed associations. We applied generalized models for location shape and scale. The differences in the associations between the T1DM group and the control group were assessed by including the respective interaction term [21]. Results were visualized using ggplot2 [22]. All statistical analyses were conducted using R version 4.2.3 [23]. The significance level was set to α = 0.05.

## Results

Table 1 presents a summary of the main characteristics and laboratory findings for our cohort and the control group. As expected, at the onset of T1DM, children had higher and abnormal values for blood glucose and HbA1c.

**Table 1:** Characteristics and laboratory values for the T1DM group at T1DM onset and the control group.

Characteristic	Control N = 1,259	T1DM N = 306	p-value
Sex			0.3
Female	569 (45%)	128 (42%)	
Male	690 (55%)	178 (58%)	
Age (years)	10.7 [7.2, 13.7]	10.3 [6.8, 13.2]	>0.068
BMI-SDS (kg/m²)	0.08 [−0.65, 0.85]	0.11 [−0.65, 0.75]	0.6
BMI group			0.3
Underweight	125 (9.9%)	31 (10%)	
Normal weight	925 (73%)	237 (77%)	
Overweight	97 (7.7%)	21 (6.9%)	
Obese	112 (8.9%)	17 (5.6%)	
Weight change during hospitalization (%)		6.6 [3.7, 10.1]	
Unknown		46	
HbA1c (%)	5.06 [4.84, 5.27]	11.20 [9.90, 13.10]	<0.001
Unknown	0	48	
Glucose (mmol/l)	5 [5, 5]	23 [18, 30]	<0.001
Unknown	145	12	
pH-value		7.35 [7.31, 7.39]	
Unknown		71	
Percentage below pH 7.35		40,85%	
hsTnT (ng/l)	3.00 [3.00, 3.89]	3.57 [3.00, 4.58]	<0.001
Perc. below LoB	693 (55%)	83 (27%)	
NT-proBNP (ng/l)	55 [31, 97]	50 [50, 69]	0,009
Perc. below LoB	31 (2.5%)	193 (63%)	

Note. Median with interquartile range in parentheses for continuous variables and counts with percentages in parentheses for categorical variables, Perc. below LoB = Percentage below the Limit of Blank.

**Fig 1:**
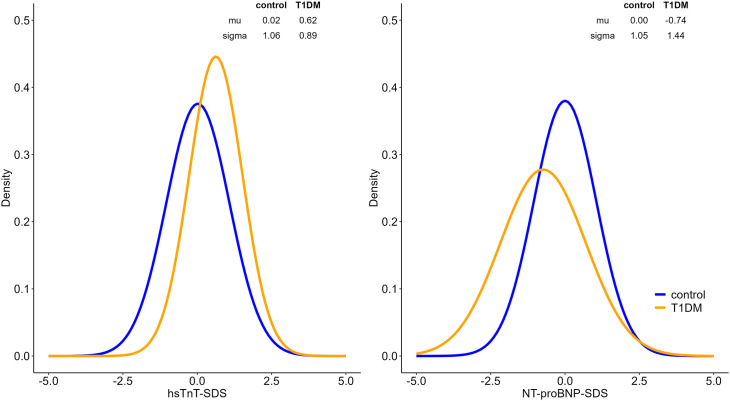
Alterations in cardiac biomarkers at time of T1DM onset compared with controls. The density plots illustrate the distribution and estimated mean (μ) and standard deviation (σ) of each biomarker in both groups. T1DM participants show a right-shifted distribution for hsTnT-SDS, indicating higher levels compared to controls, while NT-proBNP-SDS displays a broader distribution in the T1DM group.

At the onset of T1DM, children with T1DM had significantly higher TnT-SDS compared with the matched control group (ß = 0.60, p < 0.001), with a significantly lower variance in the T1DM group (ß = −0.17, p = 0.006). NT-proBNP values were significantly lower in children with T1DM (ß = −0.74, p < 0.001), with a significantly higher variance (ß = 0.31, p < 0.001) (Fig 1).

**Fig 2:**
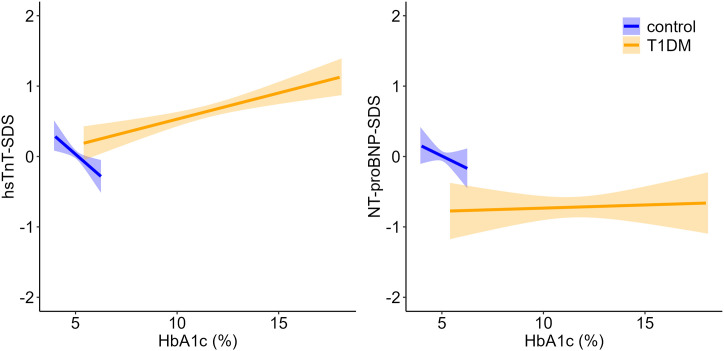
Associations between HbA1c and cardiac biomarkers (hsTnT, NT-proBNP) in children with T1DM and controls. The plots show linear regression lines with 95% confidence intervals for hsTnT-SDS (left) and NT-proBNP-SDS (right). Shaded areas indicate the uncertainty around the fitted regression. Data are stratified by group (control vs. T1DM). Data for the control group are limited to the physiological HbA1c range, resulting in a narrower confidence band.

We found a significant positive association between hsTnT-SDS and HbA1c in the T1DM group (ß = 0.07, p = 0.002), whereas a slightly negative correlation was observed in the control group (ß = −0.25, p = 0.059). However, no significant association between NT-proBNP-SDS and HbA1c was found in either group (Fig 2).

**Fig 3:**
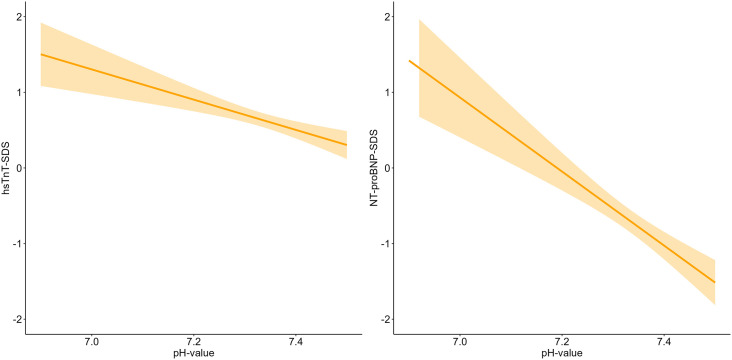
Associations between blood pH and cardiac biomarkers at T1DM onset. The plots show linear regression lines with 95% confidence intervals for hsTnT-SDS (left) and NT-proBNP-SDS (right). Shaded areas indicate the uncertainty around the fitted regression. Data are displayed across the pH range, without subgroup stratification.

In children with T1DM, hsTnT and NT-proBNP levels were significantly negatively associated with pH (ß = −0.20, p = 0.001 and ß = −0.48, p < 0.001) at the time of T1DM onset (Fig 3).

**Fig 4:**
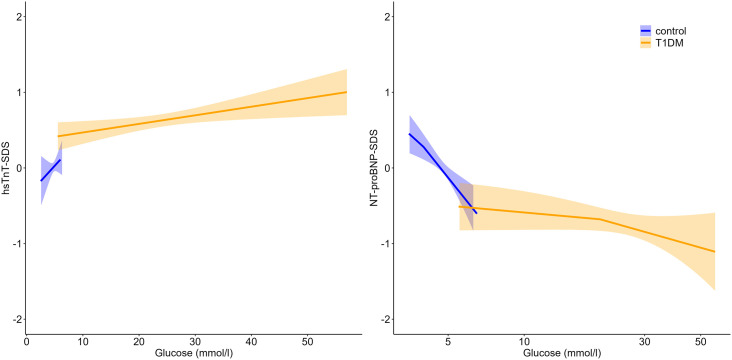
Associations between blood glucose and cardiac biomarkers at T1DM onset. The plots show linear regression lines with 95% confidence intervals for hsTnT-SDS (left) and NT-proBNP-SDS (right). Shaded areas indicate the uncertainty around the fitted regression. Data are stratified by group (control vs. T1DM).

A slightly positive trend between hsTnT-SDS and glucose levels in children with T1DM was observed (ß = 0.01, p = 0.050), this association did not reach statistical significance. No significant association was found in the control group. We found no association between NT-proBNP-SDS and glucose levels in the T1DM group. There was a negative association between NT-proBNP-SDS and glucose levels in the control group (ß = −0.35, p < 0.001) (Fig 4).

**Fig 5:**
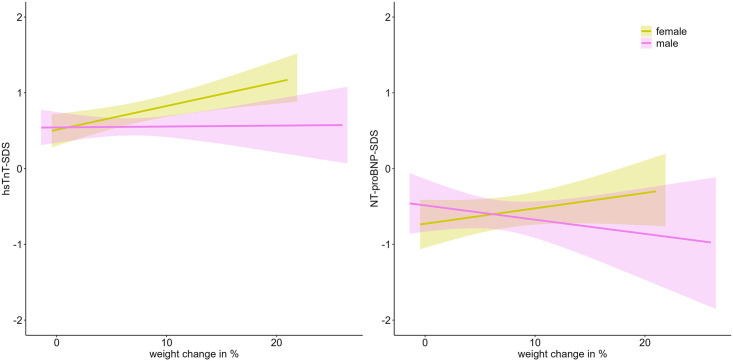
Impact of weight change between hospital admission and discharge on cardiac biomarkers at T1DM onset. The plots show linear regression lines with 95% confidence intervals for hsTnT-SDS (left) and NT-proBNP-SDS (right). Shaded areas indicate the uncertainty around the fitted regression. Data are stratified by sex. (For better visualization, we excluded two outlier values with weight change above 30%.).

In the T1DM group, a significant association between weight change and hsTnT-SDS levels was observed in female patients (ß = 0.03, p = 0.018), whereas no such association was found in male patients. No significant relationship was detected between weight change and NT-proBNP-SDS levels in either sex (Fig 5).

## Discussion

We investigated associations between cardiac markers (hsTnT and NT-proBNP) and metabolic parameters (HbA1c, pH value, and blood glucose levels) in children and adolescents at the onset of T1DM. Our results show significant differences in hsTnT and NT-proBNP levels between children at T1DM onset and a healthy matched control group without T1DM. The absolute differences were low, so it can be assumed that the timely treatment of the children at the time of onset mitigates potential cardiac damage.

### Hs-Troponin and NT-proBNP as markers for cardiovascular strain

HsTnT and NT-proBNP are established biomarkers for myocardial damage and heart failure in adults [6,7]. In our study, we found that children at T1DM onset had significantly higher hsTnT levels compared with the control group. This finding may suggest an early cardiovascular burden in children with T1DM, potentially caused by acute or chronic metabolic changes such as ketoacidosis or frequent fluctuations in blood glucose levels [24]. These results align with previous case reports of children who developed T1DM and studies of adults with T1DM, as such studies have described elevated troponin levels in connection with metabolic disorders [13,15,16,24].

Moreover, Salem et al. (2009) showed that even early in the course of T1DM, subclinical myocardial damage could be detected via LV-/RV-dysfunction and biomarkers such as NT-proBNP, highlighting the importance of monitoring cardiovascular risk from disease onset [12]. Similar results were found in adult studies showing that troponin elevation can occur even in the absence of acute coronary syndrome in cases of decompensated diabetes [14].

The significant positive association between hsTnT and HbA1c in the T1DM group (β = 0.07, p = 0.003) suggests that higher HbA1c levels are associated with increased cardiac stress, supporting the role of long-term poor blood sugar control as a risk factor for cardiovascular damage. A recent meta-analysis by Haji et al. (2023) found that T1DM is indeed associated with increased heart failure risk, further supporting our results [3]. Saeed et al. (2021) also showed that biomarkers (e.g., Galectin-3) that are associated with cardiac fibrosis and hsTnT were elevated in individuals with childhood-onset T1DM and poor glycemic control [10].

The positive association between hsTnT and HbA1c in the T1DM group gains even greater significance from the inverse association in the control group (β = −0.21, p = 0.042), which, in fact, was previously shown by our LIFE Child study group in a large cohort of healthy children [25]. It can be hypothesized that chronic hyperglycemia in diabetes mellitus may play a decisive role in the development of cardiovascular damage.

Regarding NT-proBNP levels, we found that they were significantly lower in the T1DM group compared with the control group (β = −0.72, p < 0.001), a finding that initially seems unexpected, as NT-proBNP is typically elevated in heart disease. A possible explanation could be volume depletion due to ketoacidosis, which reduces NT-proBNP production, as shown in a dehydration model by Adem et al. (2013), where BNP secretion and levels decreased under hypovolemic stress [26]. Whether long-term elevation of HbA1c levels leads to cardiac damage and elevated NT-proBNP values needs to be evaluated in further studies.

### Influence of the pH value

A notable finding was that hsTnT and NT-proBNP levels were negatively associated with the pH value in the T1DM group. The particularly strong negative association between NT-proBNP and pH (β = −0.48, p < 0.001) could point to significant effects of acute metabolic disturbances, such as the effects on cardiac function occurring in ketoacidosis. Children with severe acidosis also have higher hsTnT levels, indicating increased cardiac stress. Sakou et al. (2022) reported a case of an adolescent with severe ketoacidosis who had markedly elevated troponin and NT-proBNP levels, alongside impaired cardiac function on echocardiography [16]. From our data, it can be hypothesized that, through severe acidosis, characterized by a pH value below 7.2, cardiac strain is increased and, subsequently, higher NT-proBNP levels are observed. With a more normalized, higher pH value, other mechanisms, such as dehydration with lower BNP secretion, are likely predominant.

In line with our findings, Atabek et al. (2004) reported that children with diabetic ketoacidosis had significantly higher levels of cardiac markers such as cardiac troponin T (cTnT), creatine kinase-MB (CK-MB) and myoglobin at the time of admission compared with healthy controls. Even after 24 hours, CK-MB and myoglobin levels remained elevated, whereas cTnT levels had normalized. Notably, all markers differed significantly between patients with a pH ≥ 7.0 and those with a pH < 7.0. Furthermore, significant negative correlations were observed between pH and cTnT levels, as well as between hydrogencarbonate (HCO₃⁻) and cTnT, highlighting the importance of acid-base balance as a factor that influences myocardial stress markers. Atabek et al. reported these findings in a cohort of 19 subjects, a result we were able to replicate with our analysis of a substantially larger sample of 306 participants [15].

These results underscore the importance of closely monitoring pH values in the clinical management of children at T1DM onset and initiating early cardiac evaluation in patients at risk to prevent cardiovascular damage.

### Glucose and cardiovascular markers

There was only a slightly positive trend between hsTnT and glucose levels in children with T1DM (ß = 0.01, p = 0.052). These findings suggest that acute hyperglycemia alone might not be a primary driver of myocardial stress at the onset of T1DM. Rather, other factors (e.g., volume status or acidosis) may play a more critical role in influencing cardiac biomarker levels during this early phase [15,16]. In addition, a negative association between BNP levels and glucose was observed in the control group (ß = −0.35, p < 0.001). This relationship may be explained by several mechanisms. Slightly elevated glucose concentrations, due to hormonal differences, can induce osmotic diuresis, leading to a reduction in intravascular volume. This decrease in volume reduces atrial stretch and subsequently lowers BNP secretion [6,20,25,26]. In healthy children, BNP appears to respond more sensitively to changes in volume status than to acute metabolic fluctuations. Adem et al. (2013) demonstrated in a dehydration model that BNP levels significantly decrease under hypovolemic stress, supporting our observation [26]. BNP release is primarily stimulated by volume and pressure overload of the heart rather than by glucose levels [6].

### Weight change during hospitalization

The weight gain observed during hospitalization reflects the dimension of the initial weight loss due to dehydration, which normalizes to baseline following initiation of therapy. A significant positive association between weight gain during hospitalization and hsTnT levels was observed in girls (ß = 0.03, p = 0.018), whereas no such relationship was found in boys. The study by de Vries et al. (2023), which reported on sex-specific differences in pediatric T1DM care, showed several worse outcomes in female patients, such as higher HbA1c at diagnosis, an increased incidence of ketoacidosis, lower BMI at diagnosis, and the need for higher insulin doses during treatment [27]. These findings align with our results and suggest that girls may respond differently to early metabolic and hemodynamic changes in the disease. Differences in body composition, hormonal regulation, or inflammatory response may contribute to this sex-specific vulnerability [28]. Millstein et al. reported that over the course of the disease, women exhibit greater insulin resistance, which may be influenced by lower estrogen levels in patients with T1DM [29]. Moreover, previous studies have shown an increased risk of cardiovascular complications in female patients [30]. By contrast, no significant association was found between weight change and NT-proBNP levels in either sex, reinforcing the hypothesis that troponin and natriuretic peptides may reflect different aspects of cardiovascular stress [6,7].

### Strengths and limitations

A limitation of our study is that, in addition to standard monitoring, not all children had 12-lead ECG and echocardiography data available at the time of T1DM onset to assess heart function from an additional perspective. Furthermore, the data set contained retrospective data and therefore had some missing laboratory values. There are no comparable pH reference values available from the control group, as blood gas analysis is not part of the LIFE Child protocol for healthy participants. The great advantage of our study is that, through the diabetes registry and the Life Child cohort, there is both a reasonably large sample size and an appropriate control group from the same geographical background and time period.

## Conclusion

Our results suggest that hsTnT and NT-proBNP are useful markers for the early detection of cardiovascular strain in children and adolescents with T1DM, starting at the time of disease onset. In particular, elevated hsTnT levels, in conjunction with high HbA1c and low pH levels, could serve as an early indicator of myocardial burden and can be used to identify patients who would benefit from early cardiological assessment. NT-proBNP levels may be confounded by dehydration and hemodynamic effects and should therefore be interpreted cautiously in acute settings. Additional studies, including a longitudinal follow-up, are required to confirm these associations and investigate whether hsTnT and NT-proBNP can be used as prognostic and diagnostic markers for cardiovascular complications over the course of the disease.

## References

[1] Ogle GD, James S, Dabelea D, Pihoker C, Svennson J, Maniam J, et al. Global estimates of incidence of type 1 diabetes in children and adolescents: results from the International Diabetes Federation Atlas, 10th edition. Diabetes Res Clin Pract. 2022;183:109083. doi: 10.1016/j.diabres.2021.109083 34883188

[2] Manuwald U, Schoffer O, Kugler J, Riemenschneider H, Kapellen TM, Kiess W, et al. Trends in incidence and prevalence of type 1 diabetes between 1999 and 2019 based on the Childhood Diabetes Registry of Saxony, Germany. PLoS One. 2021;16(12):e0262171. doi: 10.1371/journal.pone.0262171 34972197 PMC8719733

[3] Haji M, Erqou S, Fonarow GC, Echouffo-Tcheugui JB. Type 1 diabetes and risk of heart failure: a systematic review and meta-analysis. Diabetes Res Clin Pract. 2023;202:110805. doi: 10.1016/j.diabres.2023.110805 37356724 PMC10530158

[4] Baden MY, Imagawa A, Iwahashi H, Shimomura I, Awata T, Ikegami H. Risk factors for sudden death and cardiac arrest at the onset of fulminant type 1 diabetes mellitus. Diabetol Int. 2015;7(3):281–8. 30603275 10.1007/s13340-015-0247-6 PMC6224989

[5] Türe M, Akın A, Unal E, Kan A, Savaş S. Electrocardiographic data of children with type 1 diabetes mellitus. Cardiol Young. 2022;32(1):106–10. doi: 10.1017/S1047951121004376 34724995

[6] Hall C. NT-ProBNP: the mechanism behind the marker. J Card Fail. 2005;11(5 Suppl):S81-3. doi: 10.1016/j.cardfail.2005.04.019 15948107

[7] Katrukha IA. Human cardiac troponin complex. Structure and functions. Biochemistry (Mosc). 2013;78(13):1447–65. doi: 10.1134/S0006297913130063 24490734

[8] Bohn MK, Steele S, Hall A, Poonia J, Jung B, Adeli K. Cardiac biomarkers in pediatrics: an undervalued resource. Clin Chem. 2021:hvab063. Available from: doi: hvab063 10.1093/clinchem/hvab063 PMC8613676 34125147

[9] Clerico A, Aimo A, Cantinotti M. High-sensitivity cardiac troponins in pediatric population. Clin Chem Lab Med. 2022;60(1):18–32. 34679265 10.1515/cclm-2021-0976

[10] Saeed M, Tapia G, Ariansen I, Stene LC, Seljeflot I, Tell GS, et al. Serum galectin-3 and subsequent risk of coronary heart disease in subjects with childhood-onset type 1 diabetes: a cohort study. Diabetes Care. 2021;44(3):810–6. doi: 10.2337/dc20-1712 33408220 PMC7896257

[11] El Razaky O, El Amrousy D, Elrifaey S, Elgendy M, Ibrahim W. Three-dimensional speckle tracking echocardiography: is it the magic wand in the diagnosis of subclinical myocardial dysfunction in children with type 1 diabetes mellitus? Echocardiography. 2018;35(10):1657–63. doi: 10.1111/echo.14095 29981180

[12] Salem M, El Behery S, Adly A, Khalil D, El Hadidi E. Early predictors of myocardial disease in children and adolescents with type 1 diabetes mellitus. Pediatr Diabetes. 2009;10(8):513–21. doi: 10.1111/j.1399-5448.2009.00517.x 19708908

[13] Poojary I, Khalid U, Patra T, Giri J, Al Heyasat A, Basith S, et al. Troponinemia in patients with diabetic ketoacidosis without acute coronary syndrome. Cureus. 2024;16(5):e61064. doi: 10.7759/cureus.61064 38915971 PMC11195327

[14] Eubanks A, Raza F, Alkhouli M, Glenn AN, Homko C, Kashem A, et al. Clinical significance of troponin elevations in acute decompensated diabetes without clinical acute coronary syndrome. Cardiovasc Diabetol. 2012;11:154. 23270513 10.1186/1475-2840-11-154 PMC3549932

[15] Atabek ME, Pirgon O, Oran B, Erkul I, Kurtoglu S. Increased cardiac troponin I concentration in diabetic ketoacidosis. J Pediatr Endocrinol Metab. 2004;17(8):1077–82. doi: 10.1515/jpem.2004.17.8.1077 15379418

[16] Sakou I-I, Soldatou A, Seretis A, Karanasios E, Paltoglou G, Karavanaki K. Markedly elevated troponin and NT-proBNP and myocardial dysfunction in an adolescent with severe diabetic ketoacidosis: a case report. Clin Pediatr Endocrinol. 2022;31(3):192–8. doi: 10.1297/cpe.2022-0017 35928382 PMC9297169

[17] Roberts KD, Levin DL. Diabetic ketoacidosis, respiratory distress and myocardial dysfunction. BMJ Case Rep. 2009;2009:bcr01.2009.1530. doi: 10.1136/bcr.01.2009.1530 PMC3028211 22132021

[18] Shim HJ, Yoo BM, Jin SM, Kang MJ. Myocardial injury in a pediatric patient with diabetic ketoacidosis: a case report. Medicine (Baltimore). 2021;100(17):e25702. doi: 10.1097/MD.0000000000025702 33907151 PMC8084016

[19] Poulain T, Baber R, Vogel M, Pietzner D, Kirsten T, Jurkutat A. The LIFE Child study: a population-based perinatal and pediatric cohort in Germany. Eur J Epidemiol. 2017;32(2):145–58. 28144813 10.1007/s10654-016-0216-9

[20] Kiess A, Green J, Willenberg A, Ceglarek U, Dähnert I, Jurkutat A. Age-dependent reference values for hs-troponin T and NT-proBNP and determining factors in a cohort of healthy children (the LIFE child study). Pediatr Cardiol. 2022;43(5):1071–83. 35277733 10.1007/s00246-022-02827-x PMC8916935

[21] Rigby RA, Stasinopoulos DM. Generalized additive models for location, scale and shape. J R Stat Soc Ser C Appl Stat. 2005;54(3):507–54.

[22] Wickham H. ggplot2: Elegant graphics for data analysis. 2nd ed. Cham: Springer International Publishing: Imprint: Springer; 2016. 1 p. (Use R!).

[23] R Core Team. R: a language and environment for statistical computing. Vienna, Austria: R Foundation for Statistical Computing; 2023. Available from: https://www.R-project.org/

[24] Kaefer K, Botta I, Mugisha A, Berdaoui B, De Bels D, Attou R, et al. Acute coronary syndrome and diabetic keto acidosis: the chicken or the egg? Ann Transl Med. 2019;7(16):397. doi: 10.21037/atm.2019.07.38 31555711 PMC6736820

[25] Kiess A, Green J, Willenberg A, Ceglarek U, Dähnert I, Kiess W, et al. Influence of growth and metabolic markers on hs-troponin T and NT-proBNP levels in healthy children. Endocr Connect. 2023;12(10):e230120. doi: 10.1530/EC-23-0120 37561076 PMC10563603

[26] Adem A, Al Haj M, Benedict S, Yasin J, Nagelkerke N, Nyberg F, et al. ANP and BNP responses to dehydration in the one-humped camel and effects of blocking the renin-angiotensin system. PLoS One. 2013;8(3):e57806. doi: 10.1371/journal.pone.0057806 23516417 PMC3596322

[27] de Vries SAG, Verheugt CL, Mul D, Nieuwdorp M, Sas TCJ. Do sex differences in paediatric type 1 diabetes care exist? A systematic review. Diabetologia. 2023;66(4):618–30. doi: 10.1007/s00125-022-05866-4 36700969 PMC9947056

[28] Maffeis C, Olivieri F, Peverelli P, Cendon M, Tomasselli F, Tommasi M, et al. Sex differences in cardiovascular risk factors of children and adolescents with type 1 diabetes mellitus: a role for diet? Nutr Metab Cardiovasc Dis. 2022;32(4):1045–54. doi: 10.1016/j.numecd.2021.11.009 35086767

[29] Millstein RJ, Pyle LL, Bergman BC, Eckel RH, Maahs DM, Rewers MJ. Sex-specific differences in insulin resistance in type 1 diabetes: the CACTI cohort. J Diabetes Complic. 2018;32(4):418–23. 10.1016/j.jdiacomp.2018.01.002 PMC5856232 29449137

[30] Brown TL, Maahs DM, Bishop FK, Snell-Bergeon JK, Wadwa RP. Influences of gender on cardiovascular disease risk factors in adolescents with and without type 1 diabetes. Int J Pediatr Endocrinol. 2016;2016:8. doi: 10.1186/s13633-016-0026-6 27099615 PMC4837565

